# Renal Function in Relation to Cardiac ^123^I-MIBG Scintigraphy in Patients with Chronic Heart Failure

**DOI:** 10.1155/2012/434790

**Published:** 2012-05-14

**Authors:** Derk O. Verschure, G. Aernout Somsen, Berthe L. F. van Eck-Smit, Hein J. Verberne

**Affiliations:** ^1^Department of Cardiology, Onze Lieve Vrouwe Gasthuis, Oosterpark 9, 1091 AC Amsterdam, The Netherlands; ^2^Department of Nuclear Medicine, Academic Medical Center, University of Amsterdam, P.O. Box 22700, 1100 DE Amsterdam, The Netherlands; ^3^Cardiology Centres of the Netherlands, IJsbaanpad 10 C, 1076 CV Amsterdam, The Netherlands

## Abstract

The aim of this study was to explore if estimates of renal function could explain variability of ^123^I-metaiodobenzylguanidine (^123^I-MIBG) assessed myocardial sympathetic activity. Furthermore estimates of renal function were compared to ^123^I-MIBG as predictors of cardiac death in chronic heart failure (CHF). Semi-quantitative parameters of ^123^I-MIBG myocardial uptake and washout were calculated using early heart/mediastinum ratio (H/M), late H/M and washout. Renal function was calculated as estimated Creatinine Clearance (e-CC) and as estimated Glomerular Filtration Rate (e-GFR). Thirty-nine patients with CHF (24 males; age: 64.4 ± 10.5 years; NYHA II/III/IV: 17/20/2; LVEF: 24.0 ± 11.5%) were studied. Variability in any of the semi-quantitative ^123^I-MIBG myocardial parameters could not be explained by e-CC or e-GFR. During follow-up (60 ± 37 months) there were 6 cardiac deaths. Cox proportional hazard regression analysis showed that late H/M was the only independent predictor for cardiac death (Chi-square 3.2, regression coefficient: −4.095; standard error: 2.063; hazard ratio: 0.17 [95% CI: 0.000–0.950]). Addition of estimates of renal function did not significantly change the Chi-square of the model. Semi-quantitative ^123^I-MIBG myocardial parameters are independent of estimates of renal function. In addition, cardiac sympathetic innervation assessed by ^123^I-MIBG scintigraphy seems to be superior to renal function in the prediction of cardiac death in CHF patients.

## 1. Introduction

The myocardial sympathetic nervous system is activated in patients with chronic heart failure (CHF) and has been shown to be associated with increased mortality. Cardiac sympathetic innervation can be scintigraphically visualized by ^123^I-metaiodobenzylguanidine (^123^I-MIBG), a radiolabelled analog of noradrenalin and has been shown to be a powerful prognostic marker in patients with CHF [1, 2]. In addition to ^123^I-MIBG there are many other prognostic markers in patients with CHF. Estimates of renal function, for example, as measured by creatinine clearance and glomerular filtration rate (GFR), have been associated with mortality and morbidity in CHF [3–5]. Interestingly in patients with chronic renal failure myocardial washout of ^123^I-MIBG, as a measure of increased myocardial sympathetic activity, has been shown to be increased [6]. However, there is limited data on a direct comparison of the respective prognostic predictive value of sympathetic hyperactivity and renal dysfunction [7]. Major clinical trials aimed to assess the prognostic value of ^123^I-MIBG have often excluded patients with substantial renal failure, further limiting the amount of prognostic information comparing these two variables [2].

Furthermore, there are complex interactions between sympathetic regulation of renal function and cardiac function. For example increased sympathetic activity reduces the renal filtration fraction [8, 9] and a reduced GFR is associated with a reduced blood clearance of ^123^I-MIBG [10]. In a recent study it was shown that differences in the rate of renal excretion did not contribute to variability in the mediastinal and myocardial ^123^I-MIBG uptake [11]. However, whether this reduced blood clearance of ^123^I-MIBG has any impact on the semiquantitative myocardial parameters is unknown. Therefore, the purpose of this study was twofold: (1) to explore if estimates of renal function could explain variability of ^123^I-MIBG assessed myocardial sympathetic activity and (2) to compare the prognostic value of estimates of renal function and myocardial ^123^I-MIBG assessed myocardial sympathetic activity in patients with CHF.

## 2. Material and Methods

The study was designed to reevaluate the results of ^123^I-MIBG imaging studies and renal function in patients with CHF prior to 1 November, 2006 in relation to cardiac events. Requirements for inclusion of subjects in this “retrospective” study were availability of the original digital ^123^I-MIBG image files; availability of serum creatinine measurements within 1 month before ^123^I-MIBG scintigraphy. Between January 1, 1996 and October 31, 2006, 39 CHF patients visiting the outpatient heart failure clinic met these requirements. Renal function was estimated using the serum creatinine-based Cockcroft-Gault equation (estimated Creatinine Clearance: e-CC) and the abbreviated MDRD equation (estimated Glomerular Filtration Rate: e-GFR) [12, 13]. Dutch national law does not require local ethics committee approval for retrospective studies. The study complies with the Declaration of Helsinki.

CHF severity was clinically evaluated according to the New York Heart Association (NYHA) classification at the time of imaging. The census date for follow-up was set at the 1 November, 2008 (at least 24 months follow-up). The mean follow-up after ^123^I-MIBG scintigraphy was 60.1 ± 37.2 months (range 1–149 months).

### 2.1. Measurement of Serum Creatinine

Serum concentrations of creatinine were determined according to routine hospital procedure. Reference levels for creatinine were 75–110 *μ*mol/L for men and 65–95 *μ*mol/L for women, respectively.

### 2.2. Renal Function

Renal function was determined by e-CC using the Cockcroft-Gault equation and expressed as mL/min:

(1) $$\begin{array}{cc} \text{e-CC} & =\frac{\left(140-\left[\text{age}\left(\text{years}\right)\right]\right)\times \left[\text{weight}\;\left(\text{kg}\right)\right]}{\left[\text{serum}\;\text{creatinine}\;\left(\mu \text{mol}/\text{L}\right)\right]}\\ & \;\times \left(1.04\;\text{for}\;\text{femals}\;\text{and}\;1.23\;\text{for}\;\text{males}\right). \end{array}$$

The e-GFR was calculated using the abbreviated MDRD equation:

(2) $$\begin{array}{cc} \text{e-GFR} & =32788\times {\left[\text{serum}\;\text{creatinine}\;\left(\mu \text{mol}/\text{L}\right)\right]}^{-1.154}\\ & \;\times {\left[\text{age}\left(\text{years}\right)\right]}^{-0.203}\times \left[0.742\;\text{for}\;\text{females}\right]\\ & \;\times \left[1.212\;\text{for}\;\text{blacks}\right], \end{array}$$

e-GFR was expressed per 1.73 m^2^ of body surface area (mL/min/1.73 m^2^). According to the guidelines for identification, management and referral of adults with chronic kidney disease, patients were stratified to an impaired kidney function (e-CC or e-GFR <60 mL/min(/1.73 m^2^)) and those with a normal e-CC or e-GFR (i.e., ≥60 mL/min/1.73 m^2^) [14].

### 2.3. ^123^I-MIBG: Acquisition and Semiquantitative Analysis

Patients underwent myocardial scintigraphy to determine ^123^I-MIBG uptake reflecting neural norepinephrine reuptake and retention. To block thyroid uptake of free ^123^I, all patients received 100 mg potassium iodide orally, one hour prior to the injection of ^123^I-MIBG. After a subsequent resting period of at least 30 minutes, patients were injected intravenously with approximately 185 MBq (5 mCi) of ^123^I-MIBG (GE Healthcare, Eindhoven, The Netherlands). Fifteen minutes (early imaging) and 4 h (delayed imaging) after MIBG administration, a 10-min planar anterior image of the thorax was acquired using a dual-head gamma-camera (e-cam, Siemens, Hoffman Estate, Illinois, USA). A 20% energy window was centred on the 159 keV photon peak of ^123^I. Images were acquired using a medium energy collimator and stored in 128∗128 matrix [15].

An experienced nuclear medicine technologist processed all planar images on a workstation (HERMES Medical Solutions, Stockholm, Sweden). The analysis of the myocardial scintigraphy data was performed blind to clinical status and estimates of renal function. ^123^I-MIBG myocardial activity was measured using a manually drawn region of interest (ROI) around the LV. The positioning of the fixed mediastinal ROI was standardized in relation to the lung apex, the lower boundary of the upper mediastinum, and the midline between the lungs [16]. To evaluate ^123^I-MIBG myocardial uptake, the Heart/Mediastinum (H/M) ratio was calculated from the early (early H/M) and delayed images (late H/M). Myocardial ^123^I-MIBG washout (WO) was defined as the percentage of change in activity from the early and delayed images:

(3) $$\left\{\frac{\left(\text{early}\;\text{H}/\text{M}-\text{late}\;\text{H}/\text{M}\right)}{\text{early}\;\text{H}/\text{M}}\right\}\times 100\%.$$

### 2.4. Follow-Up

The primary outcome was defined as cardiac death during follow-up (aggregated from: death due to acute pulmonary oedema, progressive heart failure, myocardial infarction, or ventricular arrhythmia). The secondary outcome was defined as potentially lethal ventricular arrhythmias during follow-up: documented episode of spontaneous sustained ventricular tachycardia (>30 s) ventricular tachyarrhythmia, resuscitated cardiac arrest, or appropriate ICD discharge (antitachycardia pacing or defibrillation). Long-term follow-up data were obtained from at least one of three sources: visit to the outpatient clinic; review of the patient's hospital records; personal communication with the patient's physician. An experienced cardiologist reviewed source documents to confirm occurrence of events. The cardiologist was blinded for both the estimates of renal function and the ^123^I-MIBG scintigraphic data.

## 3. Statistics

Mean values were tested for differences using the unpaired *t*-test. Linear regression was used to examine the relationship between the estimates of renal function (e-CC and e-GFR) and the ^123^I-MIBG scintigraphic data (i.e., early H/M, late H/M and washout). The overall goodness of fit was expressed as the adjusted *R* ^2^. The *F*-test was used to assess whether the model explained a significant proportion of the variability. A significant adjusted *R* ^2^ would indicate that variation in the scintigraphically determined parameters could be explained by a percentage (adjusted *R* ^2^) of change in estimates of renal function. Multivariate Cox proportional hazard regression analysis was used to investigate the relation between survival and the following parameters: age, gender, several CHF variables, estimates of renal function and the ^123^I-MIBG scintigraphic data. First, several CHF variables (left ventricular ejection fraction (LVEF), NYHA class, QRS duration) and ^123^I-MIBG semiquantitative myocardial parameters (i.e., early H/M, late H/M and myocardial washout) were entered into the model according a stepwise forward likelihood ratio-based method. Secondly, the possible additional value of renal function (e-CC and e-GFR) was determined. These data were added to the first model according the enter method (forced addition to the model). Chi-square, Cox proportional hazard regression coefficient (coefficient B), and exponent (exponent B) were used to describe the model and relative contribution of the parameters to the model. Exponent B is the predicted change in hazard for a unit increase in the predictor (i.e., hazard ratio). A *P* value < 0.05 was considered to indicate statistical significance. All statistical analyses were performed with SPSS (SPSS for Windows, version 16.0, SPSS Inc, Chicago, Il, USA).

## 4. Results

Thirty-nine patients with CHF were included in this study; all patients had stable CHF. Baseline characteristics are described in Table 1. Twenty-three patients (59%) had ischemia-related CHF and sixteen patients had nonischemic CHF. Patients with ischemia-related CHF had a lower LVEF compared to those with nonischemic CHF (*P* = 0.034). The majority was male (62%) with a mean age of 64.4 ± 10.5 years. At baseline 94.9% of patients were treated with loop diuretics, 82.1% were on angiotensin converting enzyme (ACE) inhibitor or angiotensin receptor blocker (ARB), and 46.2% were on beta-blockers.

### 4.1. ^123^I-MIBG and Estimates of Kidney Function

The mean early H/M ratio was 1.61 ± 0.46, the mean late H/M was 1.43 ± 0.38 and the mean washout was 10.1 ± 10.4% (Table 2). There was no difference in the ^123^I-MIBG semiquantitative parameters or in the e-CC and e-GFR between ischemic and nonischemic related CHF.

There were 17 patients with an impaired renal function based on e-CC (39.5 ± 10.5 mL/min, range 17–56 mL/min) and 23 with an impaired renal function based on e-GFR (42.0 ± 11.3 mL/min/1.73 m^2^, range 17–59 mL/min/1.73 m^2^). Patients with a decreased e-CC or a decreased e-GFR did not differ in ^123^I-MIBG semiquantitative parameters compared with patients with a normal e-CC or normal e-GFR (Table 3).

The variability in any of the ^123^I-MIBG semiquantitative parameters could not be explained by either e-CC or e-GFR (Table 4). Estimates of renal function could at best explain approximately 3% of the variability of the ^123^I-MIBG semiquantitative parameters (*P* = 0.851).

### 4.2. Cardiac Death

During follow-up 6 of the 39 (15.4%) patients had a cardiac death; mean interval after ^123^I-MIBG scintigraphy to cardiac death was 22 months with a range from 4 to 54 months. All 6 patients died as a result of severe progressive heart failure. Characteristics of patient with cardiac death and survivors are described in Table 5. The cardiac deaths were more likely to have a nonischemic aetiology of heart failure (*P* = 0.022). There was a statistically not significant trend towards lower e-CC and e-GFR values for patients with cardiac death compared to survivors (e-CC 53.4 ± 20.9 versus 67.8 ± 34.5, *P* = 0.375; e-GFR 49.1 ± 15.7 versus 62.0 ± 26.6, *P* = 0.259, resp.).

**Figure 1:**
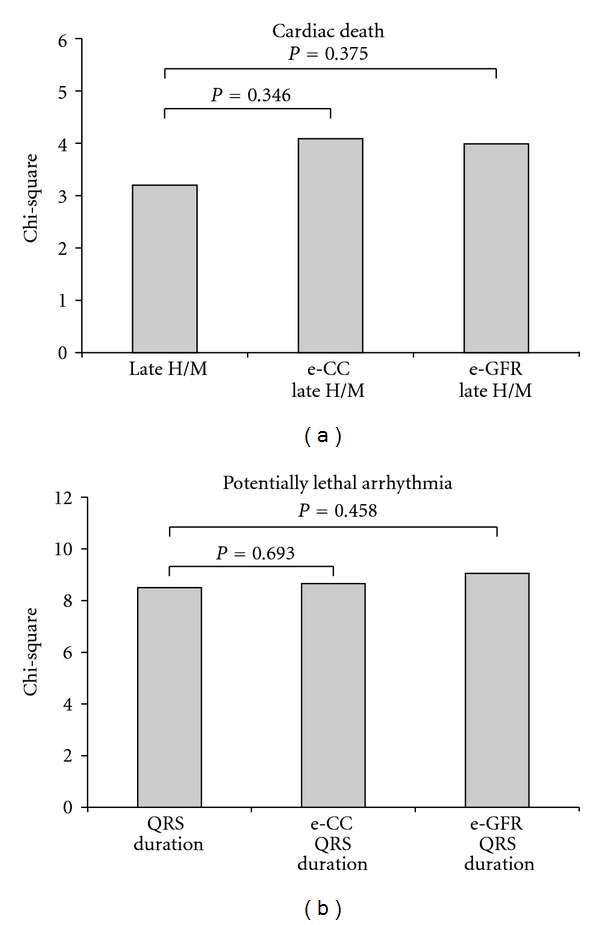
(a) Model predicting cardiac death: late H/M enters the model first (Chi-square = 3.2). The addition of renal function did not significantly change the model (Chi-square for the model including e-CC = 4.1 and for the model including e-GFR = 4.0, resp.). (b) Model predicting potentially lethal arrhythmia: QRS duration is the only significant contributor to the model (Chi-square = 8.5). The addition of renal function did not significantly change the model (Chi-square for the model including e-CC = 8.7 and for the model including e-GFR = 9.1, resp.).

Cox proportional hazard regression analysis showed that late H/M was the only independent predictor for cardiac death (Chi-square 3.2, coefficient B: −4.095; standard error: 2.063; hazard ratio: 0.17, 95% CI : 0.000–0.950). Forced addition of estimates of renal function did not significantly change the Chi-square of the model (Figure 1(a)).

### 4.3. Potentially Lethal Ventricular Arrhythmia

Nine patients developed potentially lethal ventricular arrhythmia: 5 had sustained ventricular tachycardia, 1 patient was resuscitated from a cardiac arrest, and 3 patients had an appropriate ICD discharge (i.e., antitachycardia pacing). None of these arrhythmias resulted in sudden cardiac death.

Cox proportional hazard regression analysis showed that QRS duration was the only independent predictor for a potentially lethal ventricular arrhythmia (Chi-square 8.5, coefficient B: 0.028; standard error: 0.010; hazard ratio: 1.028, 95% CI: 1.021–1.049). Forced addition of estimates of renal function did not significantly change the Chi-square of the model (Figure 1(b)). None of the ^123^I-MIBG semiquantitative parameters was predictive for a potentially lethal ventricular arrhythmia.

## 5. Discussion

Semi-quantitative ^123^I-MIBG myocardial parameters are independent of estimates of renal function. In addition, cardiac sympathetic innervation assessed by ^123^I-MIBG scintigraphy seems to be superior to renal function in the prediction of prognosis in CHF patients.

### 5.1. Renal Function and ^123^I-MIBG

In subjects with a normal kidney function, intravenous administrated ^123^I-MIBG is almost exclusively excreted via the kidneys within 24 hours after injection with approximately 35% of administered ^123^I-MIBG already excreted by 6 hours [17, 18]. As a reduced GFR is associated with a reduced blood clearance of ^123^I-MIBG, the excretion of ^123^I-MIBG is not only dependent on filtration but also by tubular secretion [10]. In short kidney function is essential for the clearance of ^123^I-MIBG and may therefore influence scintigraphic outcome. However, the results of our study show that the variability in the semiquantitative ^123^I-MIBG myocardial parameters cannot be explained by estimates of renal function. Therefore within the time frame of ^123^I-MIBG cardiac imaging (up to 4 hours after injection), the semiquantitative ^123^I-MIBG myocardial parameters are independent of renal function. These findings are in line with a recent publication showing that differences in the rate of renal excretion did not contribute to variability mediastinal and myocardial between early and late planar ^123^I-MIBG images [11]. This is eminent for clinical practice as renal dysfunction is often present in CHF patients [19, 20].

### 5.2. Renal Function, ^123^I-MIBG, and Prognosis in CHF

Renal dysfunction is not often present in patients with CHF; the serum creatinine-based estimates of renal function have been shown to be independently related to mortality [21–25]. In addition the sympathetic nervous system is one of the neurohormonal compensation mechanisms that plays an important role in the pathogenesis of CHF. Activation of this cardiac sympathetic system causes downregulation and desensitization of cardiac beta-adrenoreceptors and modification in the postsynaptic signal transduction which contributes to arrhythmia development, progression of heart failure, and ultimately cardiac death. Our results confirm previous findings that increased cardiac sympathetic activity assessed by ^123^I-MIBG scintigraphy is related to mortality [1, 2, 26].

However, there is limited data on a direct comparison of the respective prognostic predictive value of sympathetic innervation and renal dysfunction. To our knowledge only Furuhashi and Moroi studied this specific subject [7]. In patients with CHF and a preserved GFR (≥60 mL/min/1.73 m^2^) Cox proportional hazard regression analysis showed that late H/M ratio was the only independent predictor of cardiac death. However, the study lacked statistical power to perform Cox proportional hazard regression analysis in the patient group with an impaired renal function (GFR <60 mL/min/1.73 m^2^).

The lack of additional prognostic value of renal function in our study might be explained by several different but probably interacting factors. First, the aetiology of CHF differs between different studies. In studies with a larger number of patients with ischemia-related cardiomyopathy, a higher predictive value of renal function was found. This might be explained by concomitant peripheral vascular disease and secondary nephrosclerosis. Our patient cohort was not large enough to allow for adequate subgroup analysis and therefore concomitant peripheral vascular disease remains a theoretical explanation for the found discrepancies. Secondly, the differences between our results and the findings of others may be related to the prevalence of reduced kidney function. However, even in patients with increased serum creatinine levels (>2.5 mg/dL or >220 *μ*mol/L, approximately 3% of the study population), Opasich et al. were not able to identify renal function as a prognostic indicator [27]. Approximately 47% of our study population had at least a moderate impairment of renal function (i.e., e-CC or e-GFR <60 mL/min (/1.73m^2^)). This prevalence is slightly lower compared to the majority of published data. Prevalence of renal dysfunction does therefore not explain the absence of renal function as a prognostic indicator.

## 6. Limitations and Clinical Implications

The main limitation of this study is the small number of patients collected over an extended period of time when therapeutic guidelines were changing. This is reflected by the fact that the majority of included patients is relatively undertreated according to the current guidelines [28, 29]. Furthermore the mortality rate seems to be relatively low (i.e., 15%). However, the mortality rate is in line with the mortality rate as reported by other publications. Furuhashi and Moroi reported a mortality rate of 11% during a mean follow-up period of 33.7 months [7] and the cardiac mortality rate of the ADMIRE-HF study (6% during a median follow-up period of 17 months) [2]. The extrapolation of the prognostic predictive value of our study is probably influenced by these factors. The prognostic findings of our study should therefore be considered as preliminary. However, it remains that the aforementioned factors have no impact on the finding that semiquantitative ^123^I-MIBG myocardial parameters are independent of estimates of renal function.

## 7. Conclusion

Semi-quantitative ^123^I-MIBG myocardial parameters are independent of estimates of renal function. Although the findings on the prognostic predictive value of this study should be considered as preliminary, the observations suggest that cardiac sympathetic innervation assessed by ^123^I-MIBG scintigraphy is superior in the prediction of prognosis in patients with CHF to estimates of renal (dys)function. This finding might be clinically relevant as creatinine clearance is less costly to assess than ^123^I-MIBG.

## Figures and Tables

**Table 1:** Patient characteristics.

	Overall	Ischemic	Nonischemic	*P* value
	*N* = 39	*N* = 23	*N* = 16	
Age (years)	64 ± 11	66 ± 10	61 ± 11	0.962
Female/Male	15/24	6/17	7/9	0.057
NYHA class				0.351
II	17	8	9	
III	20	12	8	
IV	2	2	0	
Medical history				
Myocardial infarction	21	21	0	<0.001
CABG	8	8	0	0.008
PCI	4	4	0	0.078
Hypertension	10	5	5	0.428
Diabetes Mellitus	9	5	4	0.727
Medication				
Loop diuretics	37	22	8	0.791
ACE-I	29	17	12	0.939
ARB	3	2	1	0.778
Beta blockers	18	10	8	0.688
Amiodarone	13	9	4	0.357
Digoxin	9	4	5	0.312
Calcium channel blockers	3	2	1	0.778
LVEF (%)	24.0 ± 11.5	20.7 ± 8.6	28.6 ± 13.6	0.034
ECG				
QRS duration (msec)	163 ± 43	167 ± 36	158 ± 54	0.564
LBBB	32	21	11	0.116
RBBB	1	1	0	0.418
AF	4	2	2	0.700

NYHA class: New-York Heart association functional classification of heart failure; CABG: coronary artery bypass graft; PCI: percutaneous coronary intervention; ACE-I: angiotensin converting enzyme inhibitor; ARB: angiotensin receptor blocker; LVEF: left ventricular ejection fraction; LBBB: left bundle branch block; RBBB: right bundle branch block; AF: atrial fibrillation.

**Table 2:** Estimates of renal function and ^123^I-MIBG results.

	Overall	Ischemic	Nonischemic	*P* value
Renal function	*N* = 39	*N* = 23	*N* = 16	
e-CC	65.7 ± 33.1	58.1 ± 27.5	78.6 ± 38.6	0.076
e-GFR	60.0 ± 25.5	55.1 ± 26.6	67.1 ± 22.7	0.153
^123^I-MIBG				
Early H/M	1.61 ± 0.46	1.51 ± 0.32	1.75 ± 0.58	0.108
Late H/M	1.43 ± 0.38	1.36 ± 0.26	1.54 ± 0.49	0.139
Washout	10.1 ± 10.4	9.21 ± 10.1	11.4 ± 11.0	0.528

e-CC: estimated Creatinine Clearance; e-GFR: estimated Glomerular Filtration Rate. See for other abbreviations Table 1.

**Table 3:** Normal versus abnormal estimates of kidney function in relation to ^123^I-MIBG.

e-CC	<60 mL/min	≥60 mL/min	*P* value
	*N* = 17	*N* = 22	
Early H/M	1.45 ± 0.36	1.74 ± 0.49	0.490
Late H/M	1.29 ± 0.29	1.54 ± 0.41	0.370
Washout	9.9 ± 11.1	10.3 ± 10.0	0.915

e-GFR	<60 mL/min/1.73 m^2^	≥60 mL/min/1.73 m^2^	*P* value
	*N* = 23	*N* = 16	

Early H/M	1.57 ± 0.42	1.67 ± 0.51	0.492
Late H/M	1.38 ± 0.39	1.51 ± 0.36	0.309
Washout	11.2 ± 12.2	8.5 ± 7.0	0.432

See for abbreviations Tables 1 and 2.

**Table 4:** Variability of the estimates of renal function in relation to ^123^I-MIBG scintigraphic parameters.

	Constant	Stand error *c *	Coefficient *b *	Stand error *b *	Adjusted *R* ^2^	*P* value
e-CC versus early H/M	49.3	21.2	10.6	13.1	−0.011	0.428
e-CC versus late H/M	40.4	24.0	18.2	16.8	0.005	0.285
e-CC versus washout	66.8	8.0	−0.1	0.6	−0.029	0.851
e-GFR versus early H/M	59.1	15.4	0.6	9.2	−0.027	0.948
e-GFR versus late H/M	50.4	16.3	6.7	11.0	−0.017	0.546
e-GFR versus washout	64.8	5.7	−0.5	0.4	0.011	0.240

See for abbreviations Tables 1 and 2.

**Table 5:** Characteristics of cardiac deaths compared to survivors.

	Cardiac death	Survivor	*P* value
	*N* = 6	*N* = 33	
Age (years)	64 ± 14	64 ± 10	0.990
Female/male	2/4	13/20	0.786
NYHA class			0.529
II	2	15	
III	4	16	
IV	0	2	
Etiology			
Ischemic/nonischemic	1/5	22/11	0.022
LVEF (%)	20.8 ± 10.9	24.6 ± 11.7	0.467
ECG			
QRS duration (msec)	175 ± 66	161 ± 38	0.471
LBBB	5	27	0.647
Renal function			
e-CC	53.4 ± 20.9	67.8 ± 34.5	0.375
e-GFR	49.1 ± 15.7	62.0 ± 26.6	0.259
^123^I-MIBG			
Early H/M	1.57 ± 0.36	1.62 ± 0.47	0.839
Late H/M	1.34 ± 0.30	1.45 ± 0.39	0.512
Washout	14.2 ± 12.7	9.4 ± 9.9	0.302

See for abbreviations Tables 1 and 2.
